# Modeling the Major Influencing Factor on Match Running Performance during the In-Season Phase in a Portuguese Professional Football Team

**DOI:** 10.3390/sports10080121

**Published:** 2022-08-12

**Authors:** José E. Teixeira, Luís Branquinho, Miguel Leal, Daniel A. Marinho, Ricardo Ferraz, Tiago M. Barbosa, António M. Monteiro, Pedro Forte

**Affiliations:** 1 Research Centre in Sports Sciences, Health and Human Development (CIDESD), 5001-801 Vila Real, Portugal; 2 Department of Sport Science, Instituto Politécnico de Bragança (IPB), 5300-253 Bragança, Portugal; 3 Department of Sports, Higher Institute of Educational Sciences of the Douro (ISCE Douro), 4560-708 Penafiel, Portugal; 4 Department of Sports Sciences, University of Beira Interior (UBI), 6201-001 Covilhã, Portugal

**Keywords:** physical performance, GPS, match analysis, regression, periodization

## Abstract

The aim of this study was two-fold: (1) to analyze the influence of season phase (i.e., the start of the in-season and mid-in-season) on match running performance in a Portuguese professional football team; (2) to determine and model the main factor influencing match running performance during the in-season in this specific football team. Eighteen matches were collected by an 18 Hz global positioning system (GPS) from a professional Portuguese football team during the start of the in-season and mid-in-season. The match running performance was analyzed according to season phases, presenting significant differences in total distance (TD) (*t_lower_*_*bound*_ = 4.71, *p* < 0.001; *t_upper_*_*bound*_ = −2.22, *p =* 0.002), average speed (AvS) (*t_lower_*_*bound*_ = 359.45, *p* < 0.001; *t_upper_*_*bound*_ = −359.87, *p* < 0.001), and relative high speed running (rHSR) (*t_lower_*_*bound*_ = 13.10, *p* < 0.001; *t_upper_*_*bound*_ = −10.21, *p* < 0.001). The logistic regression showed TD (*β* = −1.59, z = −2.84, *p* = 0.005) and AvS (*β* = 2.68, z = −2.84, *p* = 0.007) as the major factors influencing match running performance during seasonal variation. Sprints and accelerations showed no significance for predicting match running performance during the season phases (*β* = −0.05 to 1.07, z = −0.95 to 1.07, *p* = 0.29 to 0.72). Current research confirms that lower and upper bounds should be used to quantify seasonal differences on match running performance. TD and AvS were described as the main factors influencing match running performance during the in-season phase. Thus, it is important to highlight the pace and volume of the game to maximize match running performance.

## 1. Introduction

Match running performance has been one of the most studied research topics in football science [1,2]. The widespread use of tracking systems has allowed an automatic quantification of physical demands, which can be extrapolated to the players’ readiness for an optimal performance [3,4]. However, match running can be influenced by seasonal variations that need to be quantified in order to model individual and collective performance [5,6]. Several studies were published demonstrating the measurement of the match load in regional [7,8], national [4,9,10], and international competitions [11,12], reporting specific seasonal variation in match running for each competition. However, only three studies were carried out in Portuguese professional, semi-professional, and amateur football leagues [7,9,10]. It is therefore important to study the Portuguese league, which is in the top 10 best European leagues and recurrently in the UEFA top-five ranking [13]. Until now, studies have focused on the influence of contextual factors and specific playing position on the match running performance of Portuguese football teams [9,10]. Barrera et al. [9] described the highest and lowest match running performance in central midfielders (10.787 ± 1536 m) and central defenders (9272 ± 455 m), respectively. In the same vein, Teixeira et al. [10] observed that the central and attacking midfielders covered a significantly greater total distance (11.54 ± 0.76 and 11.29 ± 0.55, respectively). Otherwise, attacking midfielders covered greater distances at high intensity in both studies [11,12]. The physical performance was largely influenced by the match-related contextual factors, including match location, match outcome, and opponent quality [9,11,12]. The outcome of the match was largely influenced by match running performance, with longer distances reported for winning teams (9978 ± 1963 m) or ties (10,395 ± 875 m) when compared to losing teams (9415 ± 2050) [9]. Furthermore, match running performance was influenced with trivial to very large effects by the match location and the quality of the opponent [9,10]. 

Nevertheless, the training and match load can be influenced by other factors such as season phases, type of week, players’ starting status, and training mode [2,14,15]. The influence of the season phase has already been reported before; however, it should be noted that microcycles in football tend to vary a lot between teams and game models [16,17]. Miguel et al. [7] reported that the season phase does not seem to be a factor of great influence on the match running performance in a Portuguese amateur football team. However, this evidence has not yet been reported for the professional context of Portuguese football, although there are some authors who have reported some influence of season phases on match running performance [18,19]. Hence, the current study aims to clarify this research gap by further researching the main factors that influence match running performance during seasonal variations [10]. Although some evidence on match-to-match variation in physical performance is known, the most important factor to consider has not yet been found [20,21]. Reducing the dimensionality of external load outputs generated by tracking systems remains a current challenge with high practical applicability for match running management [22,23]. Thus, the main purpose of this study was two-fold: (1) to analyze the influence of the in-season phase (i.e., the start of in-season and mid-in-season) on match running performance in a Portuguese professional football team; (2) to determine and model the major factor influencing match running performance during the in-season phase in a Portuguese professional football team. Based on assumptions from the literature, it was hypothesized that high-intensity demands were the main factors influencing match running performance during the in-season [7,10]. 

## 2. Materials and Methods

### 2.1. Participants and Match Sample

Eighteen matches were sampled from a professional Portuguese football team during the 2019–2020 in-season (Leadman LigaPro^®^, Lisbon, Portugal). A total of twenty-three male professional football players aged 32.02 ± 1.19 years were monitored. All participants signed informed consent with a description of the research aims and risks in agreement with the Declaration of Helsinki. The ethical approval was approved by the Scientific Board of the ISCE Douro under the project (ML:1;11.11.2020). 

The match data included the time–motion observations of the seven outfield players in each match of the same team (n = 128). The in-season was subdivided into two phases in agreement with the championship phases, corresponding to the matches analyzed in the first (i.e., start of in-season) and second rounds (i.e., mid-in-season) of the championship. The time–motion analysis only considered the starting players who performed the entire duration of the match, excluding those who were substituted and non-starting substitutes from the analysis [12]. The regular 90 min matches were carried out on official pitches (FIFA standard; natural grass; ~100 × 70 m), between 10:00 a.m. and 08:00 p.m. with an average environment temperature of 14.9 ± 5.3 °C [12].

### 2.2. Data Collection and Procedures

Data collection and procedures were designed according to Teixeira et al. [10]. Match data were collected using a portable 18 Hz GPS throughout the duration of the match (STATSports Apex^®^, Newry, Northern Ireland) with an accelerometer (100 Hz), magnetometer (10 Hz), and gyroscope (100 Hz). The GPS device was placed on each football player inside a custom-made mini waistcoat provided by the manufacturer on the upper back. An acceptable global navigation satellite system (GNSS) signal was receipted 30 min before the data collection, considering the standard guidelines for an optimal signal in human movement [24]. The validity and reliability of the 18 Hz GPS STATSports Apex^®^ have been well established in previous reports [24,25,26]. 

### 2.3. Match Running Performance Measures

Data for match running performance was collected with the following physical load measures according to Teixeira et al. [10]: total distance (TD) (m), average speed (AvS) (m/s), high-speed running (HSR), distance (m) (19.8–25.1 km/h), number of sprints (SPR) (>25.1 km/h), high-intensity accelerations (ACC), and decelerations (DEC) (≥3 m/s) [10]. The high-intensity thresholds for locomotor activity profile were based on standard recommendations and device specifications [2,27,28,29].

### 2.4. Statistical Analysis

Descriptive data are presented as the mean ± standard deviation (SD) with 95% confidence intervals (95% CI). Normal distribution and equal variance were tested with the Kolmogorov–Smirnov test and Levene’s test, respectively. Differences between season phases in match running performance were tested with the equivalent independent *t*-test [28] with statistical significance at *p* < 0.05. Standardized effect sizes (ESs) were calculated by Cohen’s *d* and classified as: 0.2, trivial; 0.6, small; 1.2, large; and >2.0, very large [29,30]. The major influencing factors on match running performance were computed by a multiple logistic regression analysis with an enter method [31,32]. Multicollinearity was calculated by Pearson’s partial correlations with the following magnitude: trivial if r ≤ 0.1, small if r = 0.1–0.3, moderate if r = 0.3–0.5, large if r = 0.5–0.7, very large if r = 0.7–0.9, and almost perfect if r ≥ 0.9 [30]. The model-of-fit was tested using the Akaike Information Criterion (AIC) and Bayesian Information Criteria (BIC). Pseudo R^2^ was calculated using the criteria of McFadden, Nagelkerke, Tjur, and Cox and Snell [33]. Multiple logistic regression was expressed by the estimated regression coefficients (β), standard error, z score, and Wald statistical value, representing the probability of the effect to an explanatory or independent variable [33,34]. The phase level ‘2’ (i.e., mid-in-season) was coded as class 1 (reference group). The regression line was determined using a scatter plot with density and smooth at 95% CI. All statistical analyses and data visualizations were conducted using JASP software (JASP Team, 2019; jasp-stats.org) [28].

## 3. Results

### 3.1. Match Running Performance across In-Season Phases

Table 1 presents the mean match running performance executed in each season phase (i.e., start- and mid-in-season). 

Table 2 presents the mean comparison between season phases (i.e., start of in-season vs. mid-in-season) for the match running performance. The physical load measures with significant differences amongst season phases were TD (*t_lower_*_*bound*_ = 4.71, *p* < 0.001; *t_upper_*_*bound*_ = −2.22, *p =* 0.002), AvS (*t_lower_*_*bound*_ = 359.45, *p* < 0.001; *t_upper_*_*bound*_ = −359.87, *p* < 0.001), and rHSR (*t_lower_*_*bound*_ = 13.10, *p* < 0.001; *t_upper_*_*bound*_ = −10.21, *p* < 0.001). The highest effect size was reported for rHSR (*d =* −2.06 to 2.06, moderate to very large) followed by TD (*d =* −0.61 to 0.61, moderate to very large) and AvS (*d =* −0.50 to 0.50, moderate to very large). The remaining physical load measures (i.e., SPR, ACC, and DEC) showed no significance and had trivial to small effects on the differences between the two phases of the season.

### 3.2. Main Influencing Factor on Match Running Performance during the In-Season Phase

**Figure 1:**
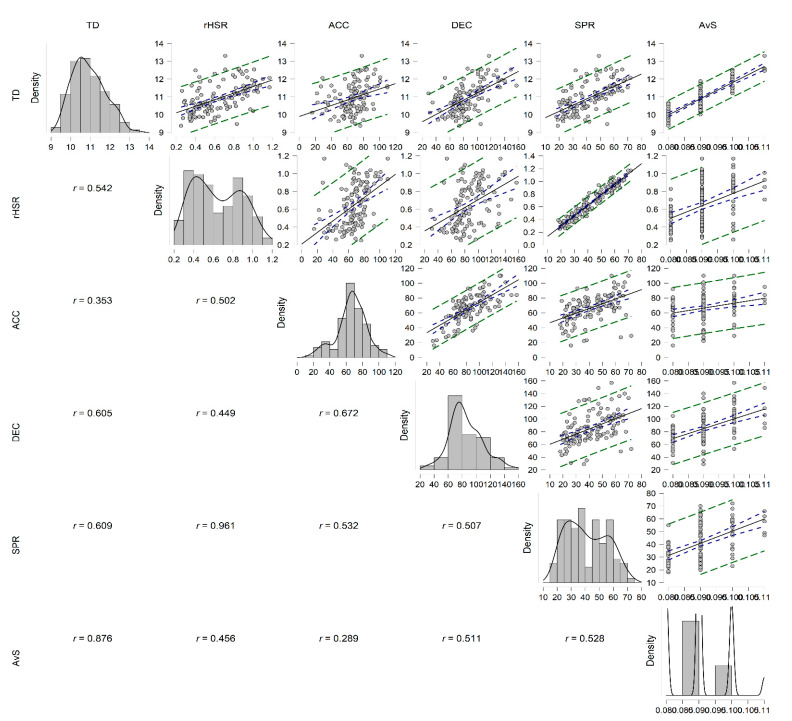
Pearson’s partial correlation between match running performance measures. Abbreviations: ACC—number of accelerations; AvS—average speed; HSR—distance at high-speed running; SPR—number of sprints; TD—total distance.

Figure 1 shows the multicollinearity between match running performance measures across season phases (i.e., start of in-season vs. mid-in-season). Positive moderate to large correlation was achieved (r = 0.353 to 0.876, *p* < 0.001). 

Table 3 presents the fit measurement to model the main factor that influences match running performance measures during the in-season phase. A good adjustment for the model was reported (AIC = 172.88, BIC = 102.84, Χ^2^ = 18.44, *p* = 0.005) whatever the applied criterion (R^2^_McFadden_ = 0.104; R^2^_Nagelkerke_ = 0.179; R^2^_Tjur_ = 0.131; R^2^_Cox & Snell_ = 0.134). 

Table 4 presents the logistic model to estimate the major factor that influences match running performance measures during the in-season phase. The logistic regression showed significance for TD (*β* = −1.59, z = −2.84, *p* = 0.005) and AvS (*β* = 1.08, z = −2.84, *p* = 0.007), with the highest magnitude shown for the first one. Regarding the high-intensity variable, physical load, HSR, SPR, ACC, and DEC showed no significance in predicting match running performance during seasonal variation (*β* = −0.05 to 1.07, z = −0.95 to 1.07, *p* = 0.29 to 0.72).

## 4. Discussion

The main purpose of this study was two-fold: (1) to analyze the influence of the in-season phase (i.e., start of in-season and mid-in-season) on match running performance in a Portuguese professional football team; (2) to determine and model the main factor that influences match running performance measures during the in-season phase in this specific football team. In general, the findings demonstrated that the in-season phase influenced the lower and upper bounds of TD, AvS, and HSR distance. TD and AvS were the main factors influencing match running performance during the in-season. It was hypothesized that high-intensity demands were the main factors influencing match running performance during the in-season; however, sprints and accelerations showed no significance in the regression analysis.

### 4.1. Match Running Performance across In-Season Phases

The influence of the season or competitive phase on match running performance has been reported in the literature with different interpretations [7,18,19]. The current research reported that the season phase does not influence the match running performance unless lower and upper limits are considered for analysis. TD, AvS, and HSR presented significant differences in the equivalent independent *t*-test (Table 2). This report is in line with the seasonal variation reported in the literature [18,19]. Malone et al. [18] reported a greater daily TD covered in the first mesocycle than in the sixth. In addition, the percentage of maximum heart rate was higher (3.3%, 1.3–5.4%) in the third mesocycle than in the first. Additionally, Jeong et al. [19] showed that the season phase influenced the weekly training load, expressed by more intense pre-season than in-season training. In the same vein, Springham et al. [35] reported a reduction trend in the match running performance over the competitive season due to the longitudinal fatigue. 

However, other research has determined the type of weekly microcycle to be a more discriminating factor in the match running performance than the season or competitive phase [2,27]. Indeed, Miguel et al. [7] described that the championship phase does not seem to be an important contributing factor to influence the external load. While the authors recognize this point, the results of the study also related higher values in TD, relative distance, and ACC for central defenders. Otherwise, attackers and wingers showed higher values for HSR distance, ACC, and DEC in the first phase than in the second phase of the championship. On the contrary, the current study demonstrated that SPR, ACC, and DEC have no significance in predicting the match running performance during the in-season phases (Table 4). These results are opposed to previous studies, as the most notable decreases in match running performance were observed in sprint performance [7,35]. Additionally, the greatest reductions in velocity and acceleration outputs were observed in full-backs, central defenders, and wingers [7,35]. The positional differences in physical performance have been extensively confirmed in the literature [4,36]. Based on the current results, season phases should not be used as the main factors influencing match running performance unless lower and upper bounds are considered. This may be due in part to the arbitrary units considered to measure the physical demands of GPS devices [2,27]. If it is impossible to individualize the high-intensity thresholds [37], the application of lower and upper bounds can partially solve the problem. This can allow more realistic inferences from the GPS data [22]. 

### 4.2. Main Factor Influencing Match Running Performance during the In-Season Phase 

Reducing the dimensionality of the GPS data has become an ongoing problem due to the large datasets generated by tracking systems [22,23]. The current research provides a good model-of-fit to measure the main factor influencing match running performance during the in-season phase. The logistic regression showed significance for TD and AvS, excluding the high-intensity variable in the prediction of the match running performance during the in-season phase. The negative estimate (β) for TD may assume a trend towards a greater collective synchronization as the season progresses [20,38]. Additionally, the positive estimate (β) for AvS could mean the demand for individual pacing strategies with individual actions are fewer in number but better in quality (search for efficiency and effectiveness) [6,23]. The psychophysiological factors should also be considered as determining issues for match running performance [3,39,40]. Regarding high-intensity demands, the logistic regression model did not consider HSR, SPR, ACC, and DEC as predictive factors for match running performance during the in-season (Figure 1). This result underlines the stability of the high-intensity demands throughout the season, although more studies are needed to prove this hypothesis. Both halves of the match were not discriminated, which may have influenced the high-intensity values [7,18,19]. The model is also limited to the physical load and season phase as independent variables. Therefore, an integrative approach of technical, tactical, and psychological factors must be taken into account for the better contextualization of the match running performance [5], considering that match running usually depends on contextual and tactical factors [9,10,38].

### 4.3. Limitations, Practical Application and Futures Perspectives

The current research showed some limitations that should be considered when interpreting the results: (i) the influence of seasonal variation (e.g., pre-season versus in-season) was not considered in the season phase comparison [41,42]; (ii) the activity thresholds were not individualized due to the non-access to raw positional data [2,27]; (iii) match data reflect only one Portuguese professional team, and therefore, the extrapolation of results to other teams and competitions should consider this. Thus, future time–motion analysis should include broader follow-up given the prospective, cross-sectional, and observational nature of the small sample size. Future research should also consider different types of weekly fixtures, players’ starting status, and the addition of complementary training sessions in weekly microcycles across season phases [2,15]. In addition, the apparent season variation in distance volume (i.e., TD) and pace capacity (i.e., AvS) must be contextualized with the team’s collective behavior and game model [5,6]. 

## 5. Conclusions

The current research confirms that lower and upper bounds must be used to quantify seasonal differences in match running performance. Applying the lower and upper bounds can partially solve the problem of the arbitrary units from the GPS data. The logistic regression analysis defined TD and AvS as the main factors that influence match running performance during the in-season phase. Thus, it is important to highlight the pace and volume of the game to maximize match running performance.

## Figures and Tables

**Table 1:** Mean match running performance according to the season phase.

Measures	Start of In-Season (Phase 1)	Mid-In-Season (Phase 2)
TD (m)	11019.0 ± 820.0	10839.0 ± 810.0
AvS (m/s)	1.49 ± 0.15	1.50 ± 0.16
HSR (m)	676.0 ± 270.0	614.0 ± 220.0
SPR (n)	43.03 ± 15.39	39.33 ± 13.15
ACC (n)	64.58 ± 20.86	68.36 ± 14.53
DEC (n)	82.61 ± 24.42	87.74 ± 22.98

Abbreviations: ACC—number of accelerations; AvS—average speed; HSR—distance at high-speed running; SPR—number of sprints; TD—total distance.

**Table 2:** Mean match running performance according to season phases.

Variables	*t*-Test			Cohen’s *d*			
Measures	Lower	*t*	Upper	Lower	*d*	Upper	Qualitative Effect
TD (m)	4.71 *	1.25	−2.22 **	−0.61	0.22	0.61	Trivial to moderate
AvS (m/s)	359.45 *	−0.21	−359.87 *	−0.50	0.83	0.50	Small to trivial
rHSR (m)	13.10 *	1.45	−10.21 *	−2.06	0.26	2.06	Moderate to very large
SPR (n)	1.66	1.47	1.27	−0.04	0.26	0.04	Trivial to small
ACC (n)	−1.04	−1.20	−1.35	−0.03	−0.21	0.03	Trivial to small
DEC (n)	−1.11	−1.22	−1.34	−0.02	−0.22	0.02	Trivial to small

Significant differences were verified as: ** *p* < 0.001; * *p* < 0.05. Abbreviations: ACC—number of accelerations; AvS—average speed; HSR—distance at high-speed running; SPR—number of sprints; TD—total distance.

**Table 3:** Fit measurement for the model of the main factor influencing match running performance during the in-season phase.

	Deviance	AIC	BIC	df	Χ^2^	*p*	McFadden R^2^	Nagelkerke R^2^	Tjur R^2^	Cox and Snell R^2^
H₀	177.321	179.321	182.173	127						
H₁	158.877	172.877	192.841	121	18.444	0.005	0.104	0.179	0.131	0.134

Abbreviations: AIC—Akaike Information Criterion; BIC—Bayesian Information Criteria; df—degrees of freedom; R^2^—Partial R squared; Χ^2^—qui-squared.

**Table 4:** Logistic model to estimate the major influencing factor on match running performance.

				Wald Test		
	Estimate (*β*)	Standard Error	z	Wald Statistic	df	*p*
(Intercept)	3.300	3.160	1.044	1.090	1	0.296
TD (m)	−1.591	0.561	−2.834	8.030	1	0.005
AvS (m/s)	1.080	1.416	2.682	7.193	1	0.007
HSR (n)	1.072	2.999	0.357	0.128	1	0.721
SPR (n)	−0.053	0.056	−0.949	0.900	1	0.343
ACC (n)	0.018	0.017	1.068	1.141	1	0.286
DEC (n)	0.023	0.014	1.714	2.937	1	0.087

Note. Phase level ‘2’ (i.e., mid-in-season) coded as class 1 (reference group for logistic analysis). Abbreviations: ACC—number of accelerations; AvS—average speed; df—degrees of freedom; HSR—distance at high-speed running; SPR—number of sprints; TD—total distance; z—z score.

## Data Availability

Data are available upon request to the contact author.
